# Knowledge, attitude, and practice regarding telemedicine among physicians and employees at Tanta University Hospitals, Egypt

**DOI:** 10.1186/s42506-025-00194-y

**Published:** 2025-06-17

**Authors:** Eman A. Younis, Amira K. El-Shenawy, Sanaa A. E. Abdo

**Affiliations:** Public Health and Community Medicine Department, Faculty of Medicine, Tanta University, Tanta, 31257 Egypt

**Keywords:** Digital health, Telemedicine, Knowledge, Attitude, Practice, Users

## Abstract

**Background:**

Telemedicine is a key factor in increasing patient accessibility, satisfaction with treatment, and quality of care, effectively utilizing physicians’ time, and improving communication among medical experts. Despite global interest in telemedicine, there is limited research exploring users’ perspectives on telemedicine within the context of Egyptian university hospitals. This study aims to examine physicians’ and employees’ levels of knowledge, attitudes, and practices toward telemedicine.

**Methods:**

A cross-sectional study was conducted at Tanta University’s medical campus from November 2023 to March 2024. One-thousand employees and physicians were surveyed. A self-administered questionnaire was used to collect the data. It consists of four sections: sociodemographic data, knowledge about telemedicine, attitude, and practice of telemedicine.

**Results:**

Half of physicians used telemedicine, and 38.2% of the employees have used e-health services. Applications of telemedicine included patients’ investigations communicated through the Internet (76.4%), patients’ management with drugs (71.4%), direct medical consultation between patient and physician (65.4%), second opinion consulting (57.6%), sharing experiences and new trends in medicine and surgery with other specialists in other countries (54%), and follow-up of patients through the electronic technologies (53%). About three-quarters of physicians and employees had a positive attitude toward telemedicine. The advantages reported include being easy to use (63%), reducing travel costs for patients (68.6%), and its importance during pandemics, e.g., COVID-19 (59.8%). However, our results indicated potential barriers when using telemedicine, including the need for training; elderly patients find difficulty dealing with technology, poor infrastructure, technical issues, difficulty for patients to express their feelings and communicate with physicians, and a lack of body language.

**Conclusion:**

A considerable percentage of participants were already using telemedicine services, and they were satisfied with the telemedicine system. Though most participants had favorable attitudes toward telemedicine, potential barriers were reported, such as training for physicians and patients, difficulty dealing with technology, poor infrastructure, and technical issues. These findings underscore the need to develop and implement a regulatory framework that supports telemedicine adoption, including data protection, patient confidentiality, and reimbursement standards.

**Supplementary Information:**

The online version contains supplementary material available at 10.1186/s42506-025-00194-y.

## Introduction

With the advent of the twenty-first century and the widespread use of social media, smartphones, personal computers, and the Internet, healthcare systems discovered that the Internet provides an excellent infrastructure for health promotion [1].

Telemedicine emerges as a response to the challenges faced by traditional healthcare systems, which are constrained by limited resources and a growing demand for services. Its goal is to increase healthcare intelligence, efficiency, and sustainability by applying advanced technologies and innovative approaches [1]. The COVID-19 pandemic has also accelerated the use of digital health innovations, including hotlines, online assistance, mobile phone apps, and many more. These technologies have played a significant role in reducing the interruption of mental health services and noncommunicable diseases, making it inevitable that digital health technology will continue [2].

Nevertheless, the health sector has had less success than other sectors in utilizing the advantages of information and communications technology applications (ICTs). Despite the available literature, there are several obstacles to overcome when putting e-health systems into place. The knowledge, acquired skills, attitude, and working environment of professionals are all important for the successful integration of any new technology [3].

Studies show higher levels of knowledge and attitudes regarding the use of telemedicine. For example, a study in India (2023) showed that most Indian healthcare workers had high scores for awareness, knowledge, and attitude toward telemedicine, but a mere 39.5% scored high for skills related to telemedicine [4]. Also, about two-thirds of Egyptian healthcare workers in 2024 exhibited good knowledge and attitude toward telemedicine. However, they reported that technical infrastructure is the main obstacle to a telemedicine application [5].

From the general population’s perspective, an Iranian study (2023) revealed that 71% of patients preferred in-person visits to telemedicine, considering more accurate diagnosis, better examination by the physician, and more accurate treatment of the disease [6]. However, an Egyptian study revealed that 35.1% of the general Egyptian population used telehealth services, and 43% expressed willingness to use them in the future. The most reported barriers were communication barriers, lack of confidence in health professionals, technological limitations, the need for physical examination, and privacy concerns [7].

The advancement of technology has made wearables, cell phones, and smaller healthcare equipment possible. Medical records can be collected using these gadgets at the patient’s residence. These gadgets will produce vast amounts of data, which artificial intelligence can integrate to enable more realistic personalized medication and more precise illness phenotyping. The medical field will become increasingly digital in the future, and acknowledging the significance of digital technology in this field and pandemic preparedness planning has become critical [8].

Digital healthcare is a broad, multidisciplinary concept that includes concepts from the intersection between technology and healthcare. Digital health applies digital transformation to the healthcare field, incorporating software, hardware, and services. Under its umbrella, digital health includes mobile health, electronic health (e-health), wearable devices, telehealth, and telemedicine, as well as personalized medicine [9].

Telemedicine (TM) is simply known as healing at a distance. This simple definition is no longer sufficient due to the sophisticated use of ICTs in many medical specializations. According to the WHO’s 2009 report, TM is defined as “the delivery of healthcare services, where distance is a critical factor, by all healthcare professionals using information and communication technologies for the exchange of valid information for diagnosis, treatment and prevention of disease and injuries, research and evaluation, and for the continuing education of healthcare providers, all in the interests of advancing the health of individuals and their communities” [10].

Telemedicine can be transferred through several ways, including transferring and storing data remotely, exchanging information between two or more clinical practitioners to aid in the diagnosis and treatment of patients who would not otherwise have timely access to specialized care [11], and telemonitoring through the use of technology such as linked medical devices and sensors for remote client/patient monitoring that allows medical professionals to keep an eye on a patient’s condition from a distance [12].

Telehealth is similar to telemedicine but includes a wide range of remote healthcare services beyond the doctor-patient relationship. It involves services provided by nurses, pharmacists, or social workers, for example, patient health education, social support, medication adherence, and troubleshooting health issues for patients and their caregivers [13].

On 29 November 2015, a new initiative to use mobile technology to control diabetes mellitus was launched in Egypt. This is a national application of the global initiative “Be He@lthy Be Mobile,” also known as “mobile health (mHealth)” [14]. On 16 March 2022, Egypt officially launched a freely accessible mental health and addiction treatment electronic platform. The digital platform provides free virtual psychoeducational services and online counseling for mental health and addiction treatment to all Egyptian citizens, including migrants and refugees [15].

Egypt has achieved significant progress in building the information society (IS) by providing an enabling legal and regulatory framework and the Information Technology Institute (ITI), Ministry of Information and Communications Technology (ICT) infrastructure. However, telemedicine projects in Egypt still face common information technology problems and challenges that hinder the wide-scale adoption of e-health systems [16].

The delivery of healthcare in impoverished nations could be entirely transformed by telehealth and telemedicine. Telemedicine has demonstrated benefits in lowering healthcare inequities, improving access to primary care services, expanding access to specialized care, and providing affordable healthcare delivery options [17]. Healthcare organizations (university hospitals in particular) affiliated with medical colleges and nursing schools are the starting point for implementing telemedicine through teaching and training their medical and paramedical graduates. Also, rising healthcare costs and a need for better treatment are motivating more hospitals to make use of the benefits of telemedicine [18].

Most studies focused on the role of health service providers, but few addressed the role of the general population as consumers. The current study focused on both, as they are the two main pillars for its application. Telehealth services are rapidly increasing, and providers’ proficiency is essential to ensuring service quality and security. Thus, it is necessary to understand professionals’ and patients’ knowledge, experience, and perspectives toward such service and identify what challenges and opportunities they experience for telehealth provision. Such identification may guide the development of telehealth education and training in health professionals’ curricula. This study aims to examine physicians’ and employees’ levels of knowledge, attitudes, and practices toward telemedicine.

## Methods

### Study design and settings

This was a cross-sectional study conducted at Tanta University’s medical campus. Tanta University is one of Egypt’s greatest and most prestigious scientific universities. It is located in the Gharbia Governorate, in the Middle Nile Delta, Egypt. Tanta University’s medical campus includes five faculties: Medicine, pharmacy, dentistry, nursing and sciences, central library, and different medical and surgical hospitals and departments providing healthcare to approximately 18 million patients yearly. This study was conducted from November 2023 to March 2024.

### Target population

- Employees working in the aforementioned five faculties and hospitals of the medical campus at Tanta University were targeted as consumers of telemedicine (being part of the general population, accessible to researchers, their work is mainly administrative and easier to investigate than patients who have limited time or have been overwhelmed with disease).
- Physicians were targeted as providers of telemedicine.

#### Inclusion criteria

- All physicians of both genders with more than 6 months of work experience at different hospitals and departments who consented to participate were included.
- All employees of the medical campus in Tanta University and hospitals who were at work during the study duration and consented to participate were included.

#### Exclusion criteria

- Medical interns and physicians with experience of less than 6 months
- Private physicians, nurses, pharmacists, and dentists

### Sample size

The sample size was calculated by using the Centers for Disease Control and Prevention, Atlanta, Georgia, USA, Epi Info 7.2.3.0 software statistical package and assuming that the expected frequency is 50%, absolute precision of 3.5%, and level of confidence of 95%. Based on the previous criteria, the sample size calculation was *N* > 784, and 20% was added to compensate for any missing data and improve validity, thus yielding 941. The authors collected 50% of the sample from physicians and 50% from employees. Researchers distributed 1050 questionnaires; 1012 were returned, 12 were excluded for missing data, and 1000 questionnaires were valid with a response rate of 95%.

### Sampling technique

The sample population was approached by convenience sampling.

### Data collection and tools of the study

Data was collected using two self-administered questionnaires (English one for the physicians and Arabic one for the employees) designed by researchers after a thorough review of the literature [7, 19–21]. The average time required to complete the questionnaire was 10–15 min.

#### First is the English questionnaire for physicians

It consists of the following:

1. Sociodemographic data, including personal information and professional background
2. Knowledge about telemedicine, which included 11 questions. Answers were scored as no = 0, uncertain = 1, and yes = 2. The total score ranged from 0 to 22. A total score of less than 50% was rated as poor, 50–75% was moderate, and > 75% was considered good knowledge. Additional multiple-choice questions about telemedicine applications, advantages, barriers, sources of information about telemedicine, and communication techniques utilized were added.
3. Attitude toward telemedicine: Assessing their opinion in 11 questions with a graded response to each statement on a three-degree Likert scale ranging from agree = 3, neutral = 2, to disagree = 1. The total attitude score ranged from 1 to 33 and was classified as follows: a total score of more than 50% was considered positive, and a score of less than 50% was considered negative.
4. Practice of telemedicine (nine questions) to evaluate the respondents’ level of skills concerning telemedicine, assessing whether they have tried telemedicine themselves, their experience, the technology used, the most common uses of telemedicine, effectiveness, and problems encountered. The authors did not score the practice.

#### The second is Arabic questionnaire for employees

It consists of the following:

1. Sociodemographic data, including personal and occupational information, and whether they had reasonable control of the Internet and electronic devices.

2. Knowledge about telemedicine, which included nine questions. Answers were scored as no = 0, uncertain = 1, and yes = 2. The score ranged from 0 to 18. A total score of less than 50% was rated as poor, 50–75% was moderate, and > 75% was considered good knowledge. Additional multiple-choice questions about telemedicine applications, advantages, and barriers to using telemedicine were added.

3. Attitude toward telemedicine: Their opinion was assessed using 10 questions with a graded response to each statement on a three-degree Likert scale ranging from agree = 3, neutral = 2, to disagree = 1. Attitude scores ranged from 1 to 30, and a score of more than 50% was considered positive.

4. Practice of telemedicine: Seventeen questions were used to evaluate whether they have tried telemedicine themselves, the frequency of using telemedicine, their experience regarding privacy, quality of service, satisfaction, and recommending telemedicine to others. The authors did not score the practice.

#### The validity of the questionnaires

The validity of the English questionnaire was assessed by three Egyptian professors from the Public Health Department at the Faculty of Medicine, Tanta University. They recommended simplifying some items and proposing very minor changes. The authors tested questionnaire reliability in a pilot study by recruiting 20 physicians not included in the present study. The internal consistency was assessed using Cronbach’s alpha. It equaled 0.832, which was adequate.

Regarding the validity of the Arabic one, it was created in English and then translated into Arabic and again into English according to WHO double-translation recommendations by English language experts. Also, the authors tested the questionnaire’s reliability in a pilot study by recruiting 20 employees not included in the present study. The internal consistency was assessed using Cronbach’s alpha. It equaled 0.793, which was adequate.

#### Data collection

The researchers and well-trained fourth-year medical students disseminated the two questionnaires at work time for employees and on different shifts for physicians.

### Statistical analysis

The collected data were analyzed using the Statistical Package for the Social Sciences (SPSS) program, version 25. Qualitative data was expressed as a number and percentage and tested using the chi-squared test. The Monte Carlo exact test was used when the chi-squared test was inappropriate. Quantitative data were expressed as median and interquartile range after testing for their normality, and the Mann–Whitney *U*-test was used. A Spearman correlation was performed between knowledge, attitude, and practice. The *p*-value was set to be significant at < 0.05.

## Results

The study included 1000 participants working in the medical campus of Tanta University; 500 employees were investigated for using telemedicine as consumers, and 500 physicians were investigated as providers. Table 1 reveals that approximately 50% of physicians used telemedicine. Of these, 69.4% mainly used text messages like WhatsApp, followed by phone calls (54.7%) and video conferences (30.6%). More than half of them used telemedicine more than twice last year (58.4%). The most commonly reported uses of e-health services in practice were for follow-up (67.3%), second opinion seeking (46.1%), and diagnostics (29.8%). Regarding the effectiveness of using e-health services in their practice, more than half (53.9%) of physicians were somewhat satisfied, and 31% were satisfied. In addition, 48.2% reported that patients committed to treatment through telemedicine to some extent, and 26.1% reported that they fully committed. Forty percent (40%) of physicians also reported problems with obtaining consent via telemedicine. Most physicians (61.2%) who did not practice telemedicine reported that they would like to try telemedicine themselves.

**Table 1:** Practice of telemedicine among the studied physicians, Tanta University Medical Campus, Egypt, 2023–2024

Questions	N = 500	
	No.	%
Have you ever used e-health services including telemedicine?		
No	255	51.0
Yes	245	49.0
If yes	(No. = 245)	
Type/media of technology used		
Text message (e.g., WhatsApp)	170	69.4
Phone call	134	54.7
Video conference	75	30.6
Personalized platform	16	6.5
Others	2	0.8
How often did you use e-health services in your practice in the last year?		
Once	55	22.4
Twice	47	19.2
More than twice	143	58.4
What are the most common uses of e-health services in your practice?^a^		
Follow-up	165	67.3
Second opinion	113	46.1
Diagnostic	73	29.8
Assisting in remote procedures	37	15.1
Others	3	1.2
What was the effectiveness of using e-health services in your practice?		
Not at all	29	11.8
Somewhat satisfactory	132	53.9
Satisfactory	76	31.0
Very satisfactory	8	3.3
Do patients commit to treatment through telemedicine?		
No	63	25.7
To some extent	118	48.2
Yes	64	26.1
Are there problems with obtaining consent via telemedicine?		
No	66	26.9
To some extent	81	33.1
Yes	98	40.0
Are there problems with clinical examination that affect your diagnosis and treatment?		
No	53	21.6
To some extent	92	37.6
Yes	100	40.8
If no	(No. = 255)	
Would you like to try telemedicine yourself?		
No	66	25.9
To some extent	33	12.9
Yes	156	61.2

^a^The percentages do not sum up to 100 due to multiple responses

Table 2 shows the characteristics of physicians in terms of their practice of telemedicine. There were no statistically significant differences regarding age, residence, income, marital status, occupation, specialty, and years of experience (*p* > 0.05). On the other hand, more than half of male physicians (54.6%) used telemedicine compared to 45.1% of female physicians, with a statistically significant difference (*p* = 0.036).

**Table 2:** Characteristics of studied physicians concerning their practice of telemedicine, Tanta University Medical Campus, Egypt, 2023–2024

Characteristics	Practice of telemedicine				Total physicians (N= 500)		*p*-value
	No (n = 255)		Yes (n = 245)				
Age (years)							
Mean ± SD	30.7 ± 6.9		31.1 ± 8.2		30.9 ± 7.5		0.502^a^
Median (IQR)	28 (27–32)		28 (26–32)		28 (27–32)		
Range	25–69		24–66		24–69		
	No	%	No	%	No	%	
Gender							
Male	94	45.4	113	54.6	207	41.4	0.036*
Female	161	54.9	132	45.1	293	58.6	
Residence							
Urban	188	51.8	175	48.2	363	72.6	0.565
Rural	67	48.9	70	51.1	137	27.4	
Income							
Not enough	91	47.9	99	52.1	190	38.0	0.102
Enough	152	54.7	126	45.3	278	55.6	
Enough and saving	12	37.5	20	62.5	32	6.4	
Marital status							
Single	121	51.7	113	48.3	234	46.8	0.789^b^
Married	129	50.0	129	50.0	258	51.6	
Widow/divorced	5	62.5	3	37.5	8	1.6	
Occupation							
Junior physicians	168	53.8	144	46.2	312	62.4	0.101
Senior physicians	87	46.3	101	53.7	188	37.6	
Specialty							
Surgical	58	44.3	73	55.7	131	26.2	0.073
Medical	197	53.4	172	46.6	369	73.8	
Years of experienc							
≤ 10 years	225	51.5	212	48.5	437	87.4	0.566
> 10 years	30	47.6	33	52.4	63	12.6	

*p*-value of chi-squared test

^a^*p*-value of Mann–Whitney U-test

^b^*p*-value of Monte Carlo exact test

^*^Statistically significant. IQR interquartile range

Table 3 demonstrates that 38.2% of employees have used telemedicine. Of these, 46.6% used it more than twice last year, 71.7% rated their experience as acceptable, and 23.6% as good. Also, 62.8% rated the time given by their healthcare provider as acceptable and 28.8% as good. Most employees reported that the physician helped them easily understand the instructions (63.4%), their privacy was respected while using telemedicine (80.6%), and they were satisfied with the physician they dealt with (69.1%) and with the doctor’s treatment (64.9%). More than half of the employees would recommend telemedicine to a friend or family member (56%). Of the employees who had never used telemedicine, 42.7% knew someone who had used telemedicine before. More than half of them (57.3%) thought telemedicine saves time and effort, and 63.1% would try telemedicine themselves.

**Table 3:** Practice of telemedicine among the studied employees, Tanta University Medical Campus, Egypt, 2023–2024

Questions	N = 500					
Have you ever used e-health services including telemedicine?	No		Yes			
	No.	%	No.	%		
	309	61.8	191	38.2		
If yes	(No. = 191)					
How often did you use e-health services in the last year?	Once		Twice		More	
	60	31.4	42	22.0	89	46.6
How to assess your experience with telemedicine?	Bad		Accepted		Good	
	9	4.7	137	71.7	45	23.6
How to assess the time your healthcare provider has given you?	Bad		Accepted		Good	
	16	8.4	120	62.8	55	28.8
	No		To some extent		Yes	
Did the doctor help you easily understand the instructions?	14	7.3	56	29.3	121	63.4
Has your privacy been respected while using telemedicine?	9	4.7	28	14.7	154	80.6
Did the team sympathize with you?	9	4.7	72	37.7	110	57.6
Were you satisfied with the doctor you dealt with?	13	6.8	46	24.1	132	69.1
Were you satisfied with the doctor’s treatment?	11	5.8	56	29.3	124	64.9
Would you recommend telemedicine to a friend or family member?	24	12.6	60	31.4	107	56.0
Have you felt been able to express yourself effectively?	23	12.0	61	31.9	107	56.1
If no	(No. = 309)					
	No		To some extent		Yes	
Do you know anyone who has used telemedicine before?	159	51.5	18	5.8	132	42.7
Do you think telemedicine provides good quality patient care?	86	27.8	143	46.3	80	25.9
Do you think telemedicine saves time and effort	39	12.6	93	30.1	177	57.3
Will you try telemedicine yourself?	107	34.6	7	2.3	195	63.1

Table 4 shows the characteristics of the employees studied in terms of their practice of telemedicine. There were no statistically significant differences between users and nonusers of telemedicine regarding age, gender, marital status, and occupation (*p* > 0.05). Regarding residence, employees from urban areas significantly used telemedicine more than those from rural areas (42.4% compared to 33.6%, respectively, *p* = 0.044). In addition, employees with “enough,” or “enough with saving income,” significantly used telemedicine more than employees with “not enough income” (45.7%, 41.9%, and 29.65%, respectively, *p* = 0.004). The percentage of using telemedicine increased with an increase in the level of education (40.4% in university education, 33.9% in secondary education, and 0.0% in primary education (*p* = 0.031). The percentage of employees using telemedicine varied significantly with the place of work (*p* = 0.031). It ranged from 25.8% of employees at the faculty of pharmacy, more than 37% of employees of Tanta University Hospitals, and the faculty of medicine to 75% of employees of the central library. Despite that, the majority of employees reported good skills for the use of the Internet and electronic devices (*n* = 444 out of 500), and 40.1% of them reported using telemedicine compared to 23.2% of employees with less control of the Internet and electronic devices, with a statistically significant difference (*p* = 0.014).

**Table 4:** Characteristics of studied employees concerning their practice, Tanta University Medical Campus, Egypt, 2023–2024

Characteristics	Practice of telemedicine				Total employees (N = 500)		*p*-value
	No (n = 309)		Yes (n = 191)				
Age (years)							
Mean ± SD	37.5 ± 10		537.5 ± 9.1		37.5 ± 10.0		0.677^a^
Median (IQR)	35 (29–45.5)		35 (31–44)		35 (30–45)		
Range	20–67		22–59		20–67		
	No.	%	No.	%	No.	%	
Gender							
Male	85	62.5	51	37.5	136	27.2	0.844
Female	224	61.5	140	38.5	364	72.8	
Residence							
Rural	158	66.4	80	33.6	238	47.6	0.044*
Urban	151	57.6	111	42.4	262	52.4	
Income							
Not enough	143	70.4	60	29.6	203	40.6	0.004*
Enough	94	54.3	79	45.7	173	34.6	
Enough and saving	72	58.1	52	41.9	124	24.8	
Marital status							
Single	40	57.1	30	42.9	70	14.0	0.680
Married	250	62.7	149	37.3	399	79.8	
Widow/divorced	19	61.3	12	38.7	31	6.2	
Educational level							
Primary	8	100.0	0	0.0	8	1.6	0.031*^b^
Secondary	78	66.1	40	33.9	118	23.6	
University	223	59.6	151	40.4	374	74.8	
Occupation							
Administrative job	144	57.6	106	42.4	250	50.0	0.062
Technical job	132	64.1	74	35.9	206	41.2	
Workers	33	75.0	11	25.0	44	8.8	
Place of work							
Faculty of medicine	76	62.3	46	37.7	122	24.4	0.031*
Faculty of pharmacy	23	74.2	8	25.8	31	6.2	
Faculty of dentistry	26	70.3	11	29.7	37	7.4	
University hospitals	164	62.6	98	37.4	262	52.4	
Central library	3	25.0	9	75.0	12	2.4	
Faculty of nursing	11	52.4	10	47.6	21	4.2	
Faculty of sciences	6	40.0	9	60.0	15	3.0	
Good use of Internet and electronic devices							
No (*n* = 56)	43	76.8	13	23.2	56	11.2	0.014*
Yes (*n* = 444)	266	59.9	178	40.1	444	88.8	

*p*-value of chi-squared test

^a^*p*-value of Mann–Whitney U-test

^b^*p*-value of Monte Carlo exact test

^*^Statistically significant. *IQR* interquartile range

Table 5 reveals a significant positive correlation between the practice of employees and physicians and their knowledge and attitude toward telemedicine (*p* < 0.05). A significant negative correlation was detected between knowledge about telemedicine and the age of employees. A significant positive correlation was also detected between the knowledge and practice of physicians and their age and years of experience (*p* < 0.05).

**Table 5:** Correlation between practices of telemedicine with knowledge, attitude, age, and years of experience among the employees and physicians

Variables	Studied employees						Studied physicians					
	Knowledge		Attitude		Practice		Knowledge		Attitude		Practice	
	*r*_s_	*p*-value	*r*_s_	*p*-value	*r*_s_	*p*-value	*r*_s_	*p*-value	*r*_s_	*p*-value	*r*_s_	*p*-value
Knowledge	-	-	0.341	0.000*	0.310	0.000*	-	-	0.395	0.000*	0.277	0.016*
Attitude	-	-	-	-	0.285	0.000*	-	-	-	-	0.266	0.021*
Age	− 0.107	0.017*	− 0.065	0.148	− 0.90	0.220	0.116	0.009*	0.059	0.185	0.360	0.002*
Years of experience	-	-	-	-	-	-	0.102	0.023*	0.053	0.235	0.253	0.029*

*r*_s Spearman rho correlation coefficient_

*Statistically significant

**Fig. 1:**
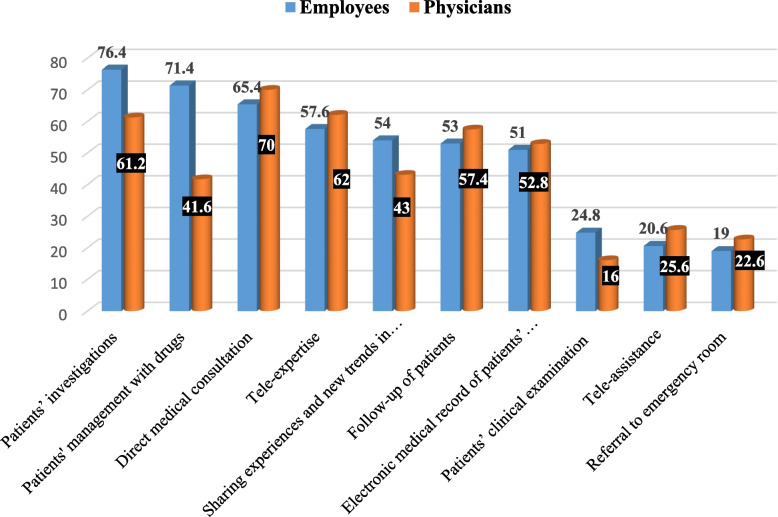
The most reported applications of telemedicine by physicians and employees, Tanta University Medical Campus, Egypt, 2023–2024

Figure 1 shows the most reported applications of telemedicine. Applications of telemedicine from the perspective of the employees included patients’ investigations communicated through the Internet (76.4%), patients’ management with drugs (71.4%), direct medical consultation between patient and physician (65.4%), second opinion consulting (tele-expertise) (57.6%), sharing experiences and new trends in medicine in surgery with other specialists in other countries (54%), and follow-up of patients through the electronic technologies (53%). For physicians, telemedicine applications included seekisecond opinion consultation (tele-expertise) (62%), communicating patients’ investigations through the Internet (61.2%), follow-up of patients through electronic technologies (57.4%), and registration of patients’ electronic medical records (52.8%).

**Fig. 2:**
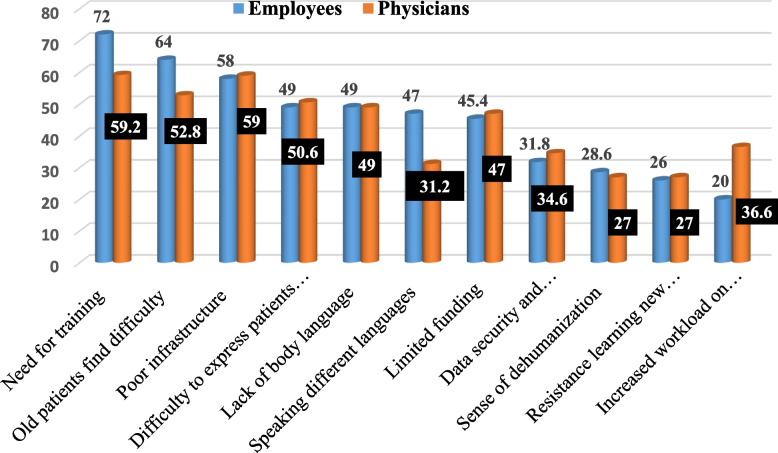
The most reported barriers to telemedicine by physicians and employees, Tanta University Medical Campus, Egypt, 2023–2024

Figure 2 reveals the barriers to telemedicine that are most reported by physicians and employees. For the employees, there was a need for training of physicians and patients (72%); some patients, especially the old ones, found difficulty dealing with technology (64%); poor infrastructure and technical issues (58%); and, lastly, the difficulty for patients to express their feelings and communicate with physicians (49%) and a lack of body language (49%). For physicians, key challenges included a need for training of physicians and patients (59.2%), poor infrastructure and technical issues (59%), and difficulty for patients to express their feelings and communicate with physicians (50.6%), particularly older patients who find difficulty dealing with technology (52.8%).

**Fig. 3:**
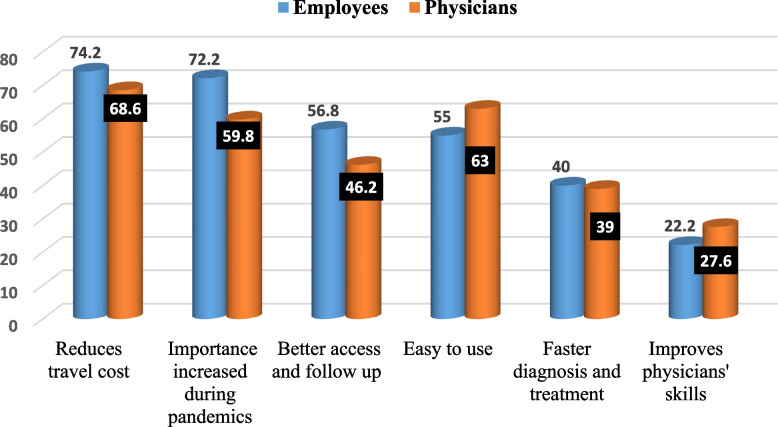
Advantages of telemedicine by physicians and employees, Tanta University Medical Campus, Egypt, 2023–2024

Figure 3 demonstrates the advantages of telemedicine. The advantages of telemedicine as perceived by the employees were that it reduces travel costs for patients (74.2%) and provides better access and follow-up for patients (56.8%), which was more evident and important during pandemics, e.g., COVID-19, and is easy to use (72.2%). The advantages of telemedicine as perceived by physicians were being easy to use (63%), it reduces travel costs for patients (68.6%), and its importance increased during pandemics, e.g., COVID-19 (59.8%).

**Fig. 4:**
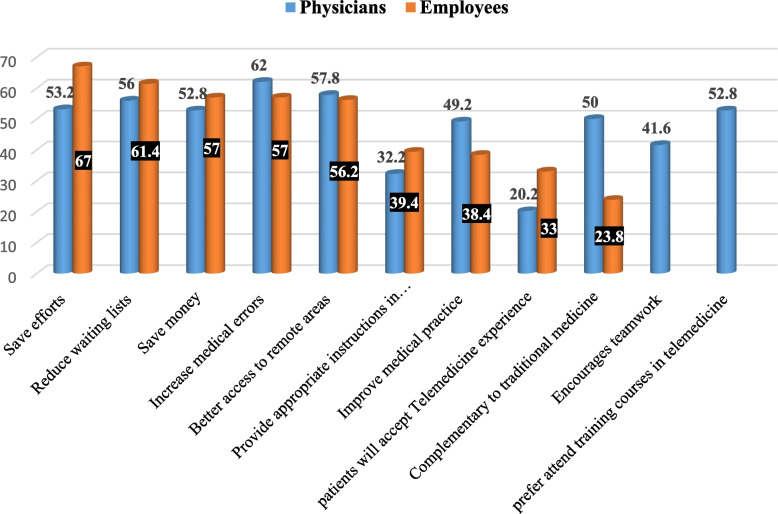
Attitudes toward telemedicine among the studied physicians and employees, Tanta University Medical Campus, Egypt, 2023–2024

Figure 4 portrays attitudes regarding telemedicine, where employees thought that telemedicine saves effort (67%), and money (57%) and provides better access to remote and underserved areas of healthcare (56.2%). On the other hand, it can increase medical errors (57%), and 43% disagreed that it can replace traditional detection and diagnosis methods. Physicians also thought positively that telemedicine saves effort (53.2%), and money (52.8%), reduces waiting lists (56%), and provides better access to remote and underserved areas of healthcare (57.8%). They also believed that traditional methods of healthcare delivery and telemedicine are complementary to each other (50%), but telemedicine can increase medical errors (62%).

Table S-1 shows the primary sources of information about telemedicine among physicians: the Internet (73.4%), colleagues (40.6%), and conferences (26%). Table S-2 illustrates that about three-quarters of both physicians and employees had a positive attitude toward telemedicine.

## Discussion

In general, various factors influence how information technology is used in healthcare organizations, just like in any other business. Appropriate tactics must be considered to address the obstacles and make technology implementation easier. Among these tactics, the human-related aspects, like users’ perceptions and knowledge of technology, are crucial. Users’ favorable opinions of technology might make information technology adoption go more smoothly and effectively [22].

### The practice of telemedicine

In the current study, approximately 50% of physicians used telemedicine. Of these, around 70% mainly used text messages like WhatsApp, followed by phone calls and video conferences. Similarly, nearly 72% of Saudi Arabian professionals always communicate with patients via social media or email [20].

During the COVID-19 pandemic, a variety of guidance and tools emerged for the scale-up of telemedicine services. Across this time span, population knowledge and learning have expanded significantly; this has led to optimizing the implementation of telemedicine services [23]. A US study revealed that 73.8% of oncologists were satisfied with the adoption of telemedicine, and after the COVID-19 pandemic, this percentage increased to 81.5% [24]. A considerable percentage of our physicians were satisfied with the telemedicine system. In agreement, 78% of the Indonesian clinicians were satisfied with the new type of care [25]. The satisfaction can be attributed to the benefits of digital health technologies, including enhanced access to secondary care advice and guidance, as well as clinical decision support tools, standardized care according to best practices, and automated checks [26]. Most of our physicians (61.2%) who did not practice telemedicine reported that they would like to use telemedicine themselves. In agreement, the majority of Saudi Arabian physicians expressed favorable opinions on telemedicine and expressed a willingness to use it in their clinical settings [20]. Also, in China, most medical professionals and staff were open to implementing and advancing telemedicine technology [27].

However, 40% of our physicians also reported problems obtaining consent via telemedicine and with the diagnosis. In concordance, a KSA study in Riyadh reported that about one-half of the doctors thought telemedicine diagnoses were incorrect because there were no physical examinations [28]. Even if technology has made it possible to preserve continuity of care, the “lack of personal touch” is still regarded as a significant disadvantage when evaluating the patient’s clinical state [29].

The current study revealed that male physicians were significantly more users than females. This higher usage among males may be attributed to their greater IT skills and, consequently, higher digital literacy. A South American study supports this suggestion, reporting that males had 3% higher IT skills than females [30].

In our study, the other sociodemographic characteristics exhibited no statistically significant differences between users and nonusers, with nearly the same percentage among both physician groups. However, physicians working in rural areas were slightly more users than urban ones. In agreement, an American study on a nationally representative random sample of 5000 family physicians in primary care showed that rural family physicians were twice as likely to use telehealth as urban family physicians. Rural physicians were more likely to use TM to take advantage of specialist expertise, expand their scope of practice, and reduce the feeling of isolation experienced by rural physicians [31].

Most users were those with “enough and saving income” and the married ones. Senior physicians with more than 10 years of experience were more users. A USA study using the 2021 National Electronic Health Records Survey (NEHRS) dataset concluded that after the COVID-19 pandemic, the majority of professionals regardless of their socioeconomic status have begun integrating telemedicine into their daily routines because, in the first place, technology enables doctors to provide care across geographic boundaries and reach a larger patient population, including those with restricted access to healthcare services. Also, doctors may serve more patients in less time, optimize scheduling, and enhance workflow by using virtual consultations to reduce the time spent on in-person visits. In addition, telemedicine may make it possible for doctors to keep an eye on their patients from a distance. This would allow for proactive and ongoing care management, which might result in early health problem diagnosis, prompt interventions, and better patient outcomes [32].

Our surgical specialists were more users than medical. In contrast, studies reported wide use of telemedicine among medical specialties as [33, 34]. However, telemedicine can be applied to surgical care in various ways. Surgeons can use telehealth to conduct pre- and postoperative surgical consultations, administer remote monitoring, and provide surgical education [35].

At the employees’ level, our results demonstrate that 38.2% of the employees have used telemedicine, and 63.1% expressed a willingness to use it in the future. In agreement, telehealth services were utilized by 35.1% of Egyptians overall, and 43% expressed willingness to use them again [7].

Our results concluded that 71.7% rated their experience with telemedicine as acceptable and 23.6% as good. An Australian survey study recorded similar findings, with 61.9% of respondents stating they had a “better” experience implementing telemedicine than the conventional medical appointment scheduling system [36].

The current results revealed that most of our employees rated the time given by their healthcare provider during a tele-visit as acceptable. Most participants reported that the doctor helped them easily understand the instructions, their privacy was respected while using telemedicine, and they were satisfied with the doctor they dealt with and the doctor’s treatment. More than half of the participants would recommend telemedicine to a friend or family member. In agreement, in a 2022 study conducted in the USA, patients’ experiences with telehealth appointments were just as good as or better than those of regular office-based visits. Tele-video visits were thought to be substantially better than office visits in terms of reports on doctor communication, care coordination, global rating, and referrals to family and friends [37]. Similarly, over 80% of Pakistani patients expressed satisfaction with their interactions with doctors via telemedicine [38]. Digital health technologies (DHTs) contribute to increased patient satisfaction and trust, particularly when dealing with sensitive medical circumstances (such as sexual or mental health difficulties) [39].

At the employee’ level, a statistically significant difference was observed between users and nonusers of telemedicine regarding residence, where urban users were more than rural. In addition, enough income and better Internet use skills had a significant effect. Our findings align with the concept of the “digital divide,” which describes the disparity between populations with and without access to contemporary communication and information technology [40].

Applications of telemedicine from the perspective of the employees included patients’ investigations communicated through the Internet, patients’ management with drugs, direct medical consultation between patient and physician, second opinion consulting (tele-expertise), sharing experiences and new trends in medicine in surgery with specialists in other countries, and follow-up of patients through the electronic technologies. In agreement, an Egyptian study reported that Egyptian patients used telemedicine mainly to follow up on their laboratory results [21]. In China, teleconsultation, remote education, telediagnosis of medical pictures, tele-electrocardiography, and telepathology were the top 5 telemedicine services used in China’s tertiary hospitals [41].

### Attitude regarding telemedicine

Our results revealed that about three-quarters of physicians had a positive attitude toward telemedicine. In concordance, the Saudi Arabian participants’ attitudes toward telemedicine were generally favorable [42]. Also, most of the Chinese study participants supported the new medical development [27].

At the employees’ level, they believed telemedicine saves efforts and money and provides better access to remote and underserved areas of healthcare. In agreement, an Egyptian study reported that more than half of the Egyptian participants thought that telehealth services may save them money and time [7]. Additionally, our results are in line with another Egyptian study conducted in the University Teaching Hospitals of eight governorates, which revealed that 73.5% of participants agreed or strongly agreed that telehealth can reduce hospital waiting lists, save time and money and transportation expenses, and provide speedier medical care [21].

### Knowledge about telemedicine

The advantages reported by physicians in this study include being easy to use, reducing patient travel costs, and increasing importance during pandemics, e.g., COVID-19. In agreement, the Indonesian clinicians reported obtaining faster diagnoses, decreasing needless referrals, enhancing the skills of healthcare professionals, and boosting patient trust [25]. In the USA, the use of preoperative telemedicine consultations in an academic medical center in Los Angeles, CA, USA, resulted in a low case cancellation rate, high patient satisfaction, and patient cost savings [43].

At the employee level, increasing accessibility through digital health technology could be a key subject, particularly for hard-to-reach groups and during pandemics, as a means to guarantee continuity of care and accessibility [39].

However, our results indicated some potential barriers when using telemedicine, including the need for training for physicians and patients; some patients, especially the old ones, find difficulty dealing with technology; poor infrastructure and technical issues; and, lastly, the difficulty for patients in expressing their feelings and communicating with physicians and a lack of body language. In agreement, the implementation of telemedicine in Libyan hospitals is adversely affected by the lack of human resources, inadequate infrastructure, training requirements, and financial concerns [19]. Also, in Saudi Arabia, the primary concerns with telemedicine adoption are patient privacy, the high expense of equipment, inadequate training, and a lack of communication between clinicians and IT specialists [20].

Poor digital literacy and the possibility that DHTs would deepen current disparities and widen the digital divide are the primary weaknesses. The best people to benefit from the increasing number of DHTs being introduced are those who live in wealthy cities and have greater levels of education, health consciousness, and digital literacy. However, underprivileged rural populations, who stand to benefit the most from remote care tools, frequently are not able to make the most of them [44].

About one-third of our participants reported barriers regarding privacy, data security, and ethical concerns. Because sensitive health data is vulnerable to hackers, digital health presents significant privacy and security challenges. The possibility of data exploitation or misuse for purposes other than healthcare was one of the ethical issues that DHTs brought. This raises concerns about data ownership and whether patient consent was obtained [45].

Our findings were in line with another study conducted in Syria, which revealed a significant positive correlation between practice, knowledge, and attitude toward telemedicine [3]. In an exploratory study of telehealth utilization among the Egyptian population (2023), telehealth use and attitude showed a statistically significant positive association, indicating that as telehealth use increased, so did positive attitudes toward telehealth services [7].

Our results show that the main sources of information about telemedicine among physicians were the Internet, colleagues, and conferences. An Iranian study (2023) concluded that the most common sources of information on telemedicine are coworkers, continuing education, social media, and the Internet [46].

### Study limitations

Our study’s cross-sectional methodology and convenience sampling technique make it impossible to draw firm conclusions about the relationships between the variables. Furthermore, our study did not include information about the various disorders that are best treated by telemedicine. Our study also does not inquire about the kinds of telemedicine technology, how they might be used, or how patients feel about telemedicine. We also conducted our research in Egypt, a developing nation. Therefore, the challenges we found might not apply to countries in other regions of the world and at various developmental stages. Further studies on telemedicine are necessary to provide patients with qualitative measurements of the quality of medical care they receive, evaluate the availability of healthcare services in other transitional nations, and determine whether these telemedicine adaptations are advantageous to patients, particularly those with mental health disorders and chronic illnesses. Additionally, it is necessary to assess whether these services require modification and to analyze their cost–benefit ratio.

## Conclusion

A considerable percentage of participants already used telemedicine services with a positive attitude, and they were satisfied with the telemedicine system. However, our results indicated some potential barriers, including the need for training for physicians and patients; some patients, especially the old ones, find difficulty dealing with technology; poor infrastructure and technical issues; and, lastly, the difficulty for patients to express their feelings and communicate with physicians and a lack of body language.

According to our findings, the perceived obstacles to adopting and continuously using e-health persist despite the continuous digital revolution. Accordingly, this finding implies that companies working on e-health innovations should prioritize offering a user-friendly interface, ideally with voice assistance, to assist users. Furthermore, we strongly advise designing patient-provider interaction apps that do not require a fast Internet connection and enable multilingual alternatives for users whose first language is not English.

Participants expressed many risk obstacles preventing the uptake and ongoing application of e-health technologies, including worries about cyberattacks, data security, patient privacy, litigation, and medical liability. To protect healthcare providers from unwarranted litigation or liability, we recommend that relevant regulatory bodies issue specific guidelines and enact appropriate statutes that align with the nature of the healthcare sector. Simultaneously, the state must charge for sufficient server space to guarantee that private information is encrypted in a centralized, government-run cloud environment without violating each healthcare organization’s data ownership.

Lastly, healthcare organizations (large hospitals in particular) affiliated with medical colleges and nursing schools should implement the required curriculum modifications to guarantee that all pre-service physicians, nurses, and other clinical staff receive the required training in the use of technology in the healthcare industry. Healthcare organizations that are implementing e-health innovations should also focus on implementation to successfully integrate their systems across all facilities and specialties. This includes ensuring that authorized access is simple, adjusting information input requirements to reduce the amount of duplicate data, and allocating sufficient resources to support continuous usage.

## Supplementary Information

Supplementary Material 1: Supplementary tables: S-1: Source of information about telemedicine among physicians. S-2: Attitude of the participants towards telemedicine

## Data Availability

No datasets were generated or analysed during the current study.
