# Supplementary material for: Knowledge, attitude, and practice regarding telemedicine among physicians and employees at Tanta University Hospitals, Egypt
Source: J Egypt Public Health Assoc. 2025 Jun 17;100:13. doi: 10.1186/s42506-025-00194-y (PMC12174010; doi:10.1186/s42506-025-00194-y)
Supplement: Supplementary file 1 — Supplementary Material 1: Supplementary tables: S-1: Source of information about telemedicine among physicians. S-2: Attitude of the participants towards telemedicine [file 42506_2025_194_MOESM1_ESM.docx]

**S-1: Source of information about telemedicine among physicians**


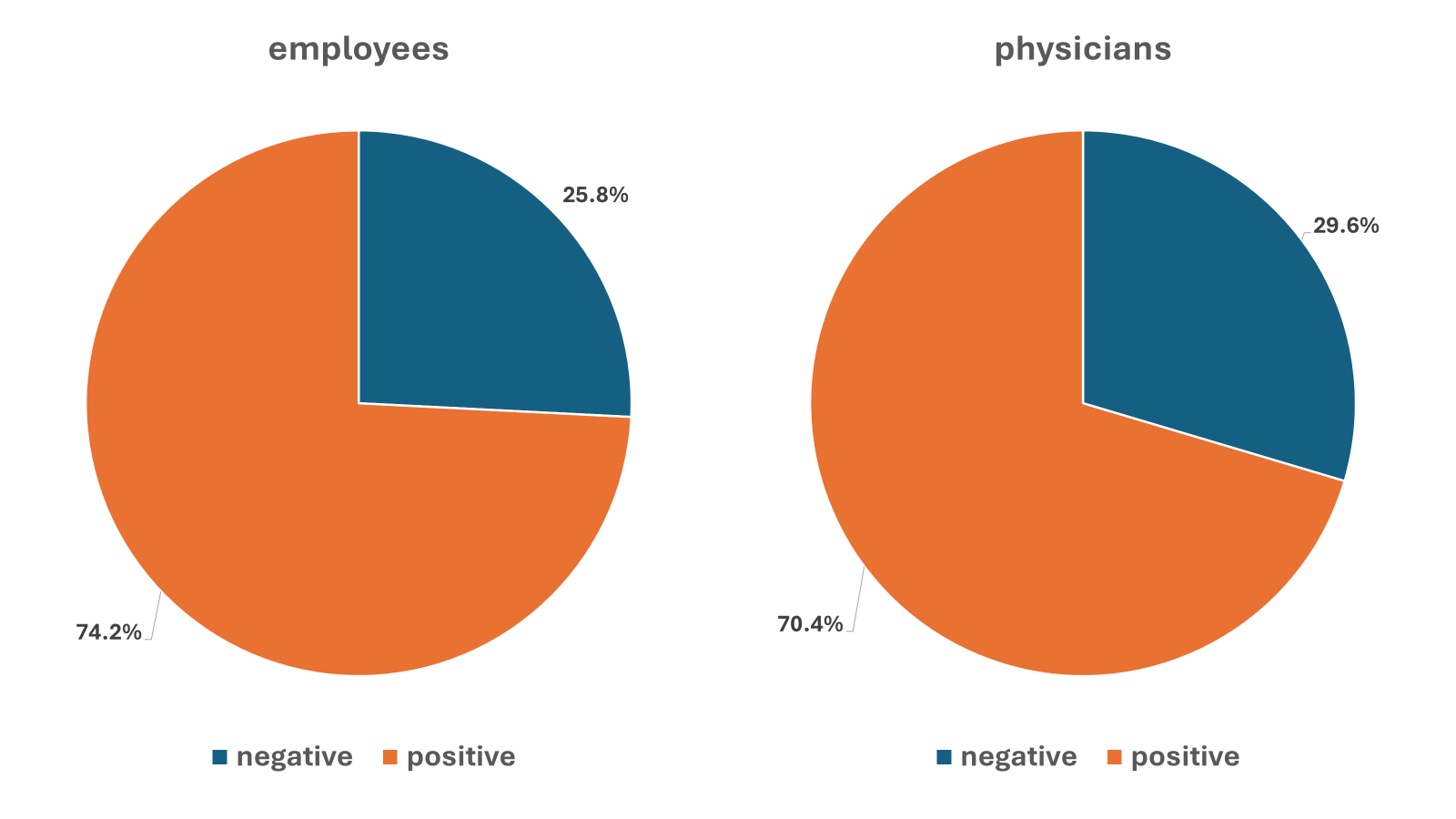


**S-2: Attitude of the participants towards telemedicine**
